# Improving Parenting Practices Among Fathers Who Misuse Opioids: Fathering Through Change Intervention

**DOI:** 10.3389/fpsyg.2021.683008

**Published:** 2021-06-21

**Authors:** Camille C. Cioffi, David S. DeGarmo

**Affiliations:** Prevention Science Institute, University of Oregon, Eugene, OR, United States

**Keywords:** parenting, opioids, fathering, intervention, behavior, substance use, misuse

## Abstract

Fathers have been largely neglected in the parenting literature though there is a critical need to improve parenting practices among fathers who misuse opioids in the midst of the opioid epidemic. Urgency is critical to rapidly intervene in the lives of fathers and children to reduce misuse and interrupt intergenerational cycles of substance misuse. Thus, we provide an overview of solutions to adapt existing parenting interventions for fathers who misuse opioids to accelerate the pace of science for this population.

## Introduction

Approximately one in eight children in the United States live with at least one parent who has a substance use disorder (Lipari and Van Horn, 2017). Problematically, it is well-documented that children exposed to parental drug use are at greater risk for maladjustment, deviant peer affiliation, and substance use initiation (Biglan et al., 2003; Dishion et al., 2003). Child welfare cases, including foster care placements and complex and severe child welfare cases, have increased in recent years and are associated with the opioid epidemic (Radel et al., 2018). Like most parenting research, much of the attention on opioid use and parenting has been focused on improving parenting among mothers who misuse opioids (Slesnick et al., 2014; Mirick and Steenrod, 2016; Gannon et al., 2017; Peisch et al., 2018; Romanowicz et al., 2019). For example, in a recent systematic review of research on the effects of parents' opioid use on children, only one study included fathers (Romanowicz et al., 2019). Given longstanding evidence that quality father involvement by residential and non-residential fathers has independent beneficial impacts on children (Aquilino, 2006; DeGarmo, 2010; Adamsons and Johnson, 2013), we argue there are benefits to understanding and improving parenting among fathers who misuse opioids. Indeed, even in the context of social disadvantage, positive father involvement is related to a host of positive outcomes for children (Cabrera et al., 2000; Gordon et al., 2012; Adamsons and Johnson, 2013; Higgs et al., 2018).

Recent work has highlighted the need to accelerate the pace of science for parents who misuse opioids in order to rapidly improve the lives of children and families affected by the rise in opioid use in the United States (Cioffi et al., 2019). To accelerate the pace of science specifically for fathers who misuse opioids, this paper describes the extant research on fathers who misuse opioids and highlights the opportunity to use and adapt existing fathering interventions to improve fathering practices among fathers who misuse opioids while simultaneously filling gaps in basic scientific knowledge about this population. Throughout this report, we broadly define fathers who misuse opioids as those who misuse a class of drugs that include heroin, synthetic opioids such as fentanyl, and pain relievers available legally by prescription, such as oxycodone, hydrocodone, codeine, morphine, and others.

## Fathering and OPIOID Use

### Importance of Father Involvement

Quality father involvement across contexts and father residency status has been associated with improved child social, emotional, behavioral, and academic outcomes (Adamsons and Johnson, 2013; Lee and Schoppe-Sullivan, 2017; Suizzo et al., 2017; Higgs et al., 2018). Involving fathers early in processes related to child welfare—including involvement in the child's case plan and enrollment in parenting, mental health and behavioral health treatment and other necessary supports to promote reunification and general father involvement—improves the father-child relationship. In turn, improved father-child relationships are related to better child behavioral health outcomes (Gordon et al., 2012). Additionally, given the high levels of co-morbid mental health and high rates of mortality experienced by parents with opioid use (Skinner et al., 2012), a father may be the only biological parent available to provide care for their child in the event that a child has been removed from custodial care of their biological mother or their mother is deceased.

### Individual Father Characteristics and Context Associated With Elevated Risk

Parents living with opioid misuse are at high risk for incarceration, family disruptions, health problems, and financial hardship (Skinner et al., 2012; Austin and Shanahan, 2017). However, research on drug abuse with men has largely neglected the role of fathering, parenting roles, and parenting status for these men (Phares, 2002; McMahon et al., 2005). Moreover, our understanding of the unique effects of opioid misuse on fathering behaviors is even more limited than our knowledge base for mothers. Within the preventive intervention and treatment research, fathers are vastly underrepresented in studies of parent training, with only a handful of studies including father-related outcomes (Panter-Brick et al., 2014). Even less is known about fathers who do or do not misuse opioids and what mechanisms are unique to opioid use that interfere with effective parenting.

The research on fathers who misuse opioids clarifies that these fathers are more likely to have experienced childhood adversity (e.g., a parent who used substances, physical abuse, foster care involvement) and early-onset substance use (Back et al., 2011; Marotta, 2017), and, if in recovery, may be facing challenges due to detoxification or withdrawal, financial instability, and family instability (e.g., marital problems, lack of family support; Bawor et al., 2015; Renk et al., 2016). Additionally, there are marked differences between men and women who misuse opioids, suggesting increased susceptibility as a function of gender. Compared to women, men are more likely to need treatment for polysubstance use and injection drug use, less likely to drop out of treatment (unless exhibiting “heavy use”), and less likely to experience psychological challenges (Back et al., 2011; Franklyn et al., 2017; Jones et al., 2017; Evans et al., 2020). Compared to mothers, fathers are more likely to be misusing opioids when they first become a parent (McMahon, 2020), and more fathers than mothers enter into drug abuse treatment (McMahon et al., 2005, 2008).

### Unstable Relationships

Fathers who misuse opioids experience a myriad of relationship challenges both with their current and previous romantic partner and their offspring. Fathers who misuse opioids report a greater prevalence of violence toward their child's mother—both over the course of the relationship and in the past year—and report greater violence perpetrated against them by their child's mother (Moore et al., 2011).

Compared to fathers without a substance use disorder, fathers who misuse opioids exhibit concerning parenting behaviors and report other limitations that may affect their children (McMahon et al., 2008). Fathers who misuse opioids report lower parental efficacy, engage in fewer positive parenting behaviors—such as consistency and positive involvement—and report less satisfaction with the parenting role (McMahon et al., 2008). Additionally, fathers with greater number of post-traumatic stress disorder symptoms engage in more problematic substance use and predict a greater frequency of negative parenting behaviors and lower frequency of positive parenting behaviors (Stover et al., 2012).

When fathers—and mothers—misuse opioids, their children are more likely to misuse opioids (Griesler et al., 2019). Moreover, in the presence of father antisocial behavior and coercive fathering, father involvement may increase a child's likelihood of engaging in antisocial behavior, leading to intergenerational transmission of antisocial behavior (Jaffee et al., 2003; Dishion et al., 2004; DeGarmo, 2010). Adolescent antisocial behavior is also associated with adolescent substance use disorders (Brennan et al., 2017). Thus, it is likely that the effects of father opioid use, antisocial behavior, coercive parenting, and social disadvantage compound and result in intergenerational transmission of substance use disorder along with other behavioral challenges. Together, these findings suggest that providing support to fathers who misuse opioids is critical to mitigating potential family disruptions and long-term consequences for children (McMahon, 2020).

### Father Services Involvement

Fathers are uniquely challenging to engage in parent training. A systematic review showed that 25% of parents in need of behavioral parent training do not enroll or engage in treatment when offered, and of those who do initially engage, 26% prematurely drop out, leaving fewer than half of the parents who had been identified as likely to benefit from behavioral parent training receiving appropriate treatment (Chacko et al., 2016). Father involvement is more limited than mother engagement in part because of approaches to engagement and training that are mother-centric (Panter-Brick et al., 2014; Parent et al., 2017). In this report, we illustrate and propose an intervention development and tailoring strategy for fathers who misuse opioids (DeGarmo, 2020).

### The Need to Accelerate Intervention Research for Fathers Who Misuse Opioids

Although there is more to be understood regarding fathering and opioid misuse, in line with recent proposals to accelerate the pace of science for parents who misuse opioids (Cioffi et al., 2019), it is prudent to apply existing fathering interventions to fathers who misuse opioids and simultaneously seek to answer basic science questions. Although there are existing effective medically-assisted treatments (e.g., methadone, naltrexone, buprenorphine) and established cognitive behavioral therapies for individuals who misuse opioids (Lam et al., 2009)—including some that have been directly tested among fathers who misuse opioids—we know of no evidence-based programs that are specifically tailored for fathers who misuse opioids.

Additionally, research is needed to understand more about the unique lived experiences of fathers with opioid use disorder and how fathers who misuse opioids may differ from; (a) mothers who misuse opioids, (b) fathers with other substance use disorders, and (c) fathers without substance use disorders. Research is also needed to understand how race and socioeconomic context intersect with these differences. Understanding the lived experiences of fathers who misuse opioids and the differences between fathers who misuse opioids and other populations will provide information on how interventions can be adapted and developed to improve fathering outcomes for fathers who misuse opioids. However, there is limited time to conduct basic research when there is an imminent need to improve outcomes for parents who misuse opioids (Cioffi et al., 2019). Thus, there are opportunities to incorporate basic science questions into intervention research and for existing fathering interventions to be adapted for fathers who misuse opioids to facilitate recovery and improve parenting practices among fathers who misuse opioids.

## Adapting and Tailoring Existing Programs For Fathers Who Misuse OPIOIDS

We have outlined a summary of considerations for tailoring existing parenting interventions to fathers with opioid misuse in Table 1. We include individual characteristics and context, father relationships, and father engagement with services. We describe recommendations for potential adaptations for fathers who misuse opioids in line with each of these considerations in the following section. We proposed that to effectively tailor these interventions for fathers, adaptation must (1) build individual capacity through a father-centric theoretical model; (2) build relationships from a strengths-based perspective, including an emphasis on the positive effect of quality father involvement; and (3) address barriers to father engagement.

**Table 1:** Parenting intervention adaptations for father who misuse opioids.

**Domain**	**Considerations**	**Potential adaptations**
Building individual capacity	• Co-morbid mental health challenges • Polysubstance use • Financial instability	• Managing stress and emotions, therapeutic support • Managing intrusive thoughts related to substance use cravings • Leveraging peer support to connect fathers to resources, provide food and housing vouchers, case management, and service navigation support
Relationship barriers	• Domestic violence • Possible separation or divorce • New, potentially unstable romantic relationships • Relationships with other people in recovery (e.g., mentor or sponsor, peers) • Father-child relationship marked by instability (e.g., removal, frequency of visits), changing norms or expectations, children at-risk for greater behavioral challenges	• Therapeutic support to rebuild relationships • Managing conflict and communication with child's other parent • Protecting child and recovery in the context of new relationships • Peer support to facilitate intervention engagement • Making the most of short visits, managing transitions, importance of consistency in parent behavior
Barriers to engagement	• Criminal justice involvement • Child welfare involvement • Behavioral treatment (inpatient or outpatient) • Standalone medication-assisted treatment (MAT)	• Balancing multiple commitments and communication • Integrating parenting skills into programming, delivered by counselor in-person or virtually or delivered on a mobile device outside of treatment hours • For MAT, short mobile-delivered sessions that can be delivered at the same time as a dose receipt

### Building Individual Capacity

First, father-centric frameworks relevant for fathers include attachment theory for fathers of infants (Ramchandani et al., 2013), social interaction and learning theory for children ages three and above (Patterson, 1982; Lewis and Lamb, 2003), and identity theory for fathers of children across the life course (Fox and Bruce, 2001; Henley and Pasley, 2005). In a county representative sample of 231 divorced fathers, for example, salience of the fathering identity and positive fathering involvement were causally associated with reductions in father substance use over time (DeGarmo et al., 2010). Although mother-centric views surrounding father substance use highlight increased stress on the mother—such as making it more difficult for the mother to quit, contributing to financial instability, and affecting the child (e.g., secondhand smoke, inadequate food or shelter, lack of father-child interaction; Magnus and Benoit, 2017)—father-centric views focus on how to increase father affiliation to their fathering role. For fathers with opioid misuse, this includes helping fathers regain control of their father identity by stimulating rewarding parent-child interactions and highlighting the positive emotional impacts of fathering on the child (Williams, 2014). In line with identity theory, enhancing the fathering role may have the benefit of shrinking father self-identification as an “addict” and reducing father substance use. To facilitate positive father self-concept, interventions must consider how to alleviate father guilt and shame about issues such as unpaid child support, prior absence, limited access to their child, previous negative or abusive interactions with their child, and their identity as an “addict” or former drug user. Additionally, fathering can play a protective role by increasing the likelihood that men will engage in treatment and sustain abstinence following treatment (Stover et al., 2011, 2018).

### Strengths-Based Relationship Building

Second, strength-based treatments increase engagement, whereas interventions based on deficit models (i.e., emphasize fathering flaws needing corrective action) are averse to fathers and threaten participation (Panter-Brick et al., 2014; Lechowicz et al., 2019). Programs that raise awareness of fathers' developmental effect on their children build father motivation. This type of approach builds rapport and trust and frames those services or treatments as a partnership working with fathers rather than working on fathers (Pfitzner et al., 2017). For example, in clinical treatments with fathers who are involved with child welfare, these fathers are responsive to interventions raising awareness of fathers' impacts on their children and interventions that emphasize the value of fathers' contributions to children's well-being (Guterman et al., 2018). This includes providing opportunities for positive parent-child interactions, supportive peer relationships, and parenting knowledge and skill acquisition opportunities (Usher et al., 2015). These differing skills will vary across the child's lifespan. For example, interventions specific to infancy may include skills related to safe infant care and bonding strategies; interventions in toddlerhood through childhood may focus on positive engagement strategies, healthy boundaries, and consistency; and in adolescence, may focus on autonomy support, building relationships, and parental monitoring.

Repairing and restoring other relationships beyond the parent-child relationship is also important for fathers in recovery. For example, attempts to reduce domestic violence and improve parenting among fathers who misuse substances have been successful (Moore et al., 2011; Stover et al., 2019).

### Addressing Barriers to Father Engagement

Finally, barriers to father engagement in parenting interventions include scheduling conflicts and timing, transportation and childcare, fatigue, motivation, stigma, and geographic location. There is a need for interventions to address barriers related to socio-economic status and system involvement (Usher et al., 2015). This may include peer supports to help fathers balance multiple commitments and offering services in multiple modalities such as web-based or in-person as well as offering services at the same time other services are being accessed. Childcare and meals may also reduce barriers to father participation, both in research and in practice.

To illustrate an approach to tailoring an existing parenting intervention to fathers who misuse opioids, we turn our attention to an adaptation of the Fathering Through Change intervention (FTC) to provide an example of tailoring and accelerating the pace of science for this population.

## Tailoring the FTC For Fathers Using OPIOIDS

Multiple parenting programs exist that show strong efficacy for improving parent self-efficacy and child behavioral outcomes [e.g., Family Check-Up (Dishion et al., 2003); Strong African American Families (Brody et al., 2006); Familias Unidas (Pantin et al., 2009; Sandler et al., 2011; Logan et al., 2014; Allen et al., 2016)]. Others have been tailored specifically for fathers, such as the Incredible Years and Triple P (Sanders et al., 2000; Webster-Stratton et al., 2004; Fletcher et al., 2011) or include fathers, such as Supporting Father Involvement and Family Foundations (Cowan et al., 2007; Feinberg et al., 2009). One example of a well-established, efficacious parent training program is Parent Management Training Oregon (PMTO). PMTO is a parent training program that has been tailored for at-risk fathers navigating the transition to the stepfather relationship (DeGarmo and Forgatch, 2007), the transition in and out of deployment cycles for military families (Gewirtz et al., 2018a,b), and for fathers navigating martial separation (DeGarmo and Jones, 2019). PMTO is recognized on numerous evidence-based practice registries and is based on social interaction learning theory, a moniker that reflects the merging of social interaction, social learning, and behavioral perspectives. Social interaction learning theory addresses ways that coercive behavioral patterns for parent-child interactions become established, maintained, and grow through reinforcing contingencies. PMTO intervention entails teaching parents how to rearrange and manage contingencies that shape children's behavior, specifically by promoting positive reinforcement of desired prosocial child behaviors and learning to eliminate coercive parenting strategies. PMTO has obtained medium to large effect sizes for observed and reported parenting behaviors and observed and reported child behaviors (DeGarmo et al., 2004; Forgatch and Patterson, 2010). FTC is a web-based adaptation of PMTO for fathers who are divorced and separated (DeGarmo and Jones, 2019). We are currently testing the feasibility of offering FTC to fathers who misuse opioids since it is web-based (i.e., suitable to the current pandemic context) and has already been tailored for fathers in particular. Thus, we consider how to tailor FTC to fathers who misuse opioids. To tailor the FTC for fathers who misuse opioids, it is critical to consider individual father characteristics and context, relationships, and engagement with services (Table 1).

### Tailoring to Build Individual Capacity

From a recovery capital perspective, substance use recovery is best understood as a contextual model that includes the “depth and breadth of internal and external resources” a parent has access to in order to initiate and sustain recovery from psychological and behavioral maladies (Kelly and Hoeppner, 2015). Simply put, recovery capital is the total sum of support resources for the recovery process. Thus, providing parenting supports independent of the fathering context is likely to be unsuccessful to achieve optimal parenting and recovery outcomes. For example, initial and ongoing engagement for fathers who misuse opioids can be particularly challenging (Lechowicz et al., 2019), therefore, there is growing integration of peer support specialists into addiction recovery services. Peer support specialists can also be trained as a coach to help problem-solve life challenges and are also effective for increasing the likelihood of abstinence (Barlow et al., 2014; Ashford et al., 2018). They can also provide connections to resources such as food and housing vouchers and case management.

Additional adaptations may include strategies to help prevent relapse in fathers with opioid misuse. A range of effective and promising treatment strategies exist to prevent relapse including Cognitive Behavioral Therapy (CBT), motivational interviewing, Twelve-Step Facilitation Therapy, community reinforcement approach, and Mindfulness-based strategies for relapse prevention (Morin et al., 2017; Ray et al., 2020). Skills taught in these evidence-based programs can be provided in modules specific to stress-reduction and craving management and principles can be embedded within modules to add depth to parent-training. For example, fathers with opioid misuse may benefit from CBT approaches to manage their anxiety symptoms triggered by stressful parenting encounters, prior to engaging with their child to address behavior.

### Tailoring to Build Relationships

Emphasizing the importance of fathers and the father-child relationship is critical to mitigate shame and guilt and increase father engagement and identity salience. This strategy may be especially important for addressing opioid abuse. Approaches that address father relationships within the whole family context, including strategies aimed at repairing relationships and addressing domestic violence, if present, increase father engagement in treatment, improve family relationships, and reduce long-term opioid misuse (Fals-Stewart and O'Farrell, 2003; Stover et al., 2019).

Peer support specialists may also be beneficial for building relationships and navigating parenting concerns for fathers with opioid misuse. Engaging a peer support specialist who has lived experience as a father may help facilitate initial intervention uptake and ongoing engagement with parentingsupports. Additionally, peer support specialists may help the father navigate other interpersonal relationships such as those with a child's other caregiver(s) and provide a bridge to additional therapeutic resources to help facilitate community and healthy relationship building.

### Tailoring to Address Barriers to Engagement

FTC for fathers in recovery should consider a variety of settings. For example, nesting delivery in a group-based setting in intensive outpatient, day treatment, or inpatient programs may promote collective support and problem-solving. Skills could also be delivered one-on-one in counseling sessions or a blend between these two approaches. However, a limitation of integration into treatment settings is that it is rare for fathers to have the same access to childcare or ability to bring their child to inpatient treatment, something which is still limited but more available for mothers. Alternatively, some fathers may be receiving medication-assisted treatment and no behavioral treatment. In these instances, brief interactions may increase engagement and mobile-based delivery may be optimal. Mobile-based delivery could also be used following treatment to reinforce skills and provide access to ongoing support.

## Conclusion

We have described the extant research on fathers who misuse opioids and used FTC as an example of how to adapt existing fathering interventions to improve fathering practices among fathers who misuse opioids while simultaneously filling gaps in basic scientific knowledge. It is prudent to direct our resources to simultaneously test existing fathering interventions among fathers who misuse use opioids and gather basic science information about this population in order to accelerate the pace of science for families affected by opioid misuse.

## Author Contributions

CC wrote the first draft of the manuscript. DD wrote sections of the manuscript. All authors contributed to manuscript revision, read, and approved the submitted version.

## Conflict of Interest

The authors declare that the research was conducted in the absence of any commercial or financial relationships that could be construed as a potential conflict of interest.
